# Trauma matters! Trauma-informed care among allied health professionals working with children and youth and its associations with personal factors

**DOI:** 10.1192/j.eurpsy.2025.689

**Published:** 2025-08-26

**Authors:** A. Stern, L. Lamash, N. Ghanem

**Affiliations:** 1 Occupational Therapy, Ben-Gurion University of the Negev, Beer Sheva; 2 Occupational Therapy, University of Haifa, Haifa, Israel

## Abstract

**Introduction:**

Childhood trauma can significantly impact development, function, and well-being. Allied health professionals often support individuals exhibiting trauma-related signs, but the extent of trauma-informed care (TIC) understanding and application remains unclear. Moreover, secondary traumatic stress (STS) is a significant concern for professionals who treat traumatized children and youth. Thus, it is essential to provide TIC training to these professionals while also addressing the potential effects of STS.

**Objectives:**

The current study mapped the knowledge of trauma, the perceived relevance of TIC, and its implementation among allied health professionals working with children and youth. It also examined the relationships between knowledge of trauma, TIC perceived relevance and implementation, and relevant personal factors (resilience, self-compassion, and empathy).

**Methods:**

176 allied health professionals (nutritionists and occupational, speech, and physical therapists) answered an online survey including a demographic questionnaire, Trauma-Informed Approach Questionnaire, Connor-Davidson Resilience Scale, Self-Compassion Scale, and Interpersonal Reactivity Index. Descriptive statistics and ANOVA were used to describe the sample and assess differences between knowledge, attitudes, and implementation. Pearson correlations were used to assess relationships with personal factors.

**Results:**

Significant differences were found between trauma knowledge, TIC relevance perception, and TIC implementation among the entire sample, *F*(2, 352) = 127.5, *p*<.001, *η²*= 0.43. Perception of TIC relevance was higher than knowledge of trauma (p<.001) and TIC implementation (p<.001). Positive correlations were found between resilience and knowledge of trauma (*r*=.22, p<.01), TIC perception relevance (*r*=.17, *p*<.05), and TIC implementation (*r*=.23, *p*<.05). Self-compassion was positively correlated with knowledge of trauma (*r*=.18, *p*<.05) and TIC implementation (*r*=.22, *p*<.01). Perspective-taking (empathy) was positively correlated with knowledge of trauma (*r*=.15, *p*<.05), perception of TIC relevance (*r*=.39, *p*<.01), and TIC implementation (*r*=.31, *p*<.01), and empathic concern was positively correlated with perception of TIC relevance (*r*=.33, *p*<.01).

**Conclusions:**

The findings highlight the limited knowledge of trauma and TIC implementation among allied health professionals, emphasizing the lack of TIC training. Resilience, self-compassion, and empathy can be strategies to cope with treating children and youth who have experienced trauma and prevent STS.

**Disclosure of Interest:**

None Declared

